# Hospital and Institutional News

**Published:** 1914-03-21

**Authors:** 


### HOSPITAL AND INSTITUTIONAL NEWS.

### RADIUM AND THE CANADIAN GOVERNMENT.

Simultaneous with the announcement that a Government Bill has been introduced into the Provincial Legislature, offering a reward of £5,000 for the first discovery of radium in the Province of Ontario, Canada, Mr. Borden is stated by the Ottawa correspondent of the *Times* to have declared that an Order in Council has been passed reserving to the Crown the right in any radium that may be discovered in Canada. A bonus to encourage prospecting is also contemplated, and we hope that these steps may have the double effect of showing Canada to be no less rich in ore than her neighbour, and of securing to the hospitals an available supply without the inflation of prices which the commercial exploitation of radium necessitates. In view of the present state of the market, as our present issue shows, these developments are likely to be carefully watched in this country.

### ISOLATION HOSPITAL FLOODED OUT.

The extraordinary rainfall of last week, culminating in a week-end of gale and storm, has had serious effects in many parts of the country, and the isolation hospital for East and West Molesey has been one of the worst sufferers. The several blocks of which the institution is composed have been converted into islands owing to the depth of water which surrounded them, and which was so deep that the medical officer was unable to approach the institution in his motor-car, and had to fall back upon the higher, if less convenient, farm carts, in which he forded the road to the hospital. All the staff were similarly incommoded, passage from one block to another being possible only in wading-boots, while the matron is said to have made her rounds on a trolley, which is sufficient evidence of the depth of the water. Esher was cut off from Hersham, so far as the main road was concerned, and in the main street of the village of West Molesey itself the water attained a height of over three feet, to the dismay of the inhabitants, whose houses were with very few exceptions under water. While the school had to be closed because of the flood, the isolation hospital and its staff have, we are glad to learn, been able to pursue their work, under difficulties which, if far from agreeable at the time, will make a memory to laugh over, and one that will require no effort to keep alive.

### MOLIERE ON THE NURSING HOME.

We have published a sufficient number of articles giving the patient's point of view of the nursing home to show that there is a wide public capable of appreciating, objectively, the comedy of an exposé of the evils complained of on the stage. But it would be hardly fair to the author of "The Rest Cure," a one-act play, dealing with the comedy of the nursing home, which was produced at the Vaudeville Theatre last Monday, to say that this alone accounted for the welcome which it received. Miss Gertrude Jennings is becoming an old hand at the one-act play, and was breaking new ground very happily in her latest picture of a man of letters suffering from nerves (endemic, we believe, in America) undergoing a month's rest-cure in the quiet of a "home," where the traffic outside vied in disturbance with the conscientious breaking of every rule which Florence Nightingale laid down as the basis of nursing. Her terse dictum: "Windows are made to open, doors are made to be shut," was indeed carried out in the sense that the doors in this home were never doing anything else; but fun is also extracted, as was inevitable, if less true to life, from the conduct of the nurses. Most of all, indeed, the character of the maid-of-all-work, whose keen sense of fact and grip both of the needs of invalids and the habits of commercial enterprises, which prey on them in the form of using their hypochondria as an excuse for doubling the prices of the most pretentious hotel, while providing none of its "luxuries," provides the Molière element of comedy in the piece. True comedy, the most intellectual of the arts, has always taken high rank, because its laughter cleanses without bitterness and reveals without sensationalism, and the present little piece deserves well of the hospital world, because a certain amount of genuine observation has gone to its making, and there is no exploitation of the operating theatre, surgical or electrical "discoveries," —and much excellent fun.

### A HOSPITAL STRIKE IN ROME.

A hospital is very rarely, if ever, made the occasion of a strike, but such has been the case in Rome, where, owing to the condemnation of the old San Giacomo Hospital for Incurables, and its prompt closing, a strike took place on the part of the populace to show their sympathy with some employees who were thus thrown out of work. The strike was sufficiently widespread to stop the tramcars and to cause the shops to remain closed. The police were reinforced by soldiers in the patrol of the streets, which was necessary for a period of two days. Peace was restored by the reopening of a few beds in San Giacomo, and an inquiry into the staff's grievances was held. Of greater importance, however, is the determination to build an important hospital, to consist of 600 beds, in seven pavilions for surgical cases, four for medical, and also an isolation block. The site chosen is on the slope of Monteverde, a congested quarter of Rome. In addition the Technical Hospital Office has approved the addition of a new floor to the Consolation Hospital and the erection of an infectious diseases hospital for 250 beds. In the result, therefore, the strike has further directed public attention to the need for modernising the hospital accommodation in Rome.

### THE RECONSTRUCTION OF BROWNLOW HILL WORKHOUSE.

The proposal to reconstruct Brownlow Hill Workhouse referred to in our last issue has given rise to much discussion in the Liverpool press.

It is pointed out that many thousands of pounds have been spent on the old buildings in recent years in the provision of new sanitary towers, of improved ventilation, and of an operating theatre, and the Local Government Board is blamed for allowing this expenditure and for not insisting upon a new building fifteen or twenty years ago. There is some ground for this complaint, for it has been obvious for years past that no tinkering with the building could render it suitable for use as an infirmary for acute cases. But this should have been realised by the members of the Liverpool Select Vestry themselves. The cost of the new building is variously estimated from one quarter to a million pounds. The actual cost would probably be somewhere between these two estimates. A modern hospital for 1,200 patients can hardly be built for less than half a million. It is said that this is too much to expect Liverpool to pay; but the bill is presented to-day as the result of Liverpool's neglect in the past, and there is no excuse for postponing further the day of reckoning. A matter worthy of more consideration has, however, been introduced into the discussion.

### THE FUTURE POOR-LAW UNIT.

It is said that the institutions of the three Liverpool Unions—Liverpool, West Derby, and Toxteth—have 3,500 vacant places even in the busiest time of the year, and that at other times this number would probably reach 5,000. It is suggested that by an amalgamation of the three Unions suitable accommodation could be found for all the inmates of the Brownlow Hill Institution without any need for fresh building. If these statements are correct a very strong case has been made out for amalgamation. The advantages are obvious. There would be a great saving in the cost of administration, greater efficiency would be obtained as the result of the improved classification rendered possible, and the Brownlow Hill site could be sold, or the buildings on it, by a comparatively inexpensive scheme of reconstruction, be used for healthy inmates and cases of chronic disease. Similar schemes of amalgamation have already been carried out in several areas, notably in Birmingham and in London. In the Birmingham area the Unions of Birmingham, Aston, and King's Norton were amalgamated recently, with the result that there has been a great improvement in the efficiency of the provision made for the sick poor. In London the areas involved have been smaller, and the results, therefore, less striking. There can be no doubt that the future of Poor-Law administration lies in the formation of large areas which will permit of the provision and proper staffing of specialised institutions.

### A POISON MUSEUM.

Only a fortnight ago we reviewed a book on the subject of murder mysteries, and pointed out the increasing part which medical and scientific testimony now plays in the disentanglement of criminal mysteries. This aspect of medico-legal studies has been officially recognised at University College Hospital by the opening of a museum at the medical school. The specimens include varieties of poisons from prussic acid to pyradin, and tissues showing the effects of their absorption. In addition, to illustrate the contrast of the poisons of decomposition with those directly due to the introduction of extraneous substances, *post-mortem* specimens are included, together with examples of bloodstains on knives, metal, cloth, and wood, side by side with the effects of rust and other stains on similar material. The exhibits also include examples of gunshot wounds, and a beginning has been made towards the cataloguing of the signs of death, by placing side by side contrasted examples of the behaviour of the tissues under normal and abnormal circumstances, together with the substances employed in causing death, whether by physical or chemical agency.

### THE COUNTY COUNCIL AND MENTAL DEFICIENCY.

The London County Council has appointed its first committee for administering the Mental Deficiency Act, and we sincerely trust that the members of that body appreciate the importance of the work which lies before them. It is for the Council to see that in carrying out these new functions they rise above all questions of party, uniting in a common effort for the welfare of the community. Every year party seems to be playing a greater part in the work of the Council, but there has always been one notable exception, which encourages us to hope for the best with regard to the mental deficiency work. The Mental Hospitals Committee is one of the very few committees of the Council where political considerations are left out of the question, and where each member strives to do his best for the unfortunate people who come under his care. There is little glamour about the work. The members of the committee rarely come into the limelight. They are for the most part engaged either at County Hall or at the hospitals in all parts of the county and outside it. It is possibly because the visible glory to be derived from the work is very small that there is no overwhelming anxiety to serve on the Mental Hospitals Committee. At the present moment it is six short of its proper strength. By standing orders it should consist of thirty members; the most desperate efforts have failed to induce more than twenty-four members of the Council to serve upon it, and it is admitted that some of these are nominal members who take little part in the routine work. At the present time the whole work of the committee is really borne on the shoulders of twenty members of the Council. Possibly the novelty of the new committee may attract some fresh members to it, but there is a grave danger that unless the Council realises its great responsibility the same difficulty may arise as with the Mental Hospitals Committee.

### THE PARENT OF NURSING HOMES.

The mushroom growths of recent years in the direction of so-called nursing homes lends a special interest to the pioneer institution which set the example thirty-six years ago, and has continued with increasing prosperity to supply hospital treatment for those of comparatively small means—namely, Fitzroy House. At the annual meeting of the Home Hospitals Association in the Fitzroy Square institution on Monday, Sir John Bland Sutton, who presided, pointed out that the past year had been the most satisfactory they had ever had in the history of the Association. No fewer than 482 patients had been admitted, whose average length of stay was seventeen days; eighty-three medical men had sent patients; the mortgage had been reduced to £4,300; and there was a balance in hand of £891. The additional theatre, constructed two years ago, had justified its cost, and no increase in the patients' fees had been resorted to. Sir Bland Sutton referred also to the death of Mr. Frederic Cox, who had been treasurer from 1879 to 1912, and who was also one of the trustees. It is exceedingly satisfactory to know that the Home Hospitals Association continues successfully to maintain the high standard which it was the first to set up, and of which commercial competition has been the worst guarantee, as the public and the authorities are at last beginning to understand. The Fitzroy House record is a fine one, and shows what can be done where wise administration has been combined with the intention really to supply the missing link between free treatment and treatment of which the cost is not considered.

### A MEDICAL OFFICER'S CLAIM TO A PENSION.

A remarkable claim for a superannuation allowance was successfully sustained by Dr. James Damer Priest, late district medical officer and public vaccinator for the Waltham Abbey District of Edmonton Union, in an action before Mr. Justice Horridge and a special jury in the King's Bench Division last week. The plaintiff's case was that he held the above offices from 1886 till January 1912, receiving an average salary of £105, from which deductions under the Acts relating to the superannuation of Poor-Law officers were annually made by the Edmonton Guardians. From 1905 he suffered from nyctalopia, which in 1911 developed into a permanent infirmity, by which he was rendered incapable of performing his duties, which he resigned on January 1, 1912. The defendants thereupon passed a resolution granting him a superannuation allowance of £53 5s. 7d. until July 1913, when the resolution was rescinded and payment discontinued. He therefore claimed the allowance as due from the latter date. The defendants denied permanent incapacity, and evidence was brought forward to show that the plaintiff had a large panel practice and was doing other work. The plaintiff in the witness-box stated that he was able to do the work of a district medical officer by day, and where roads were adequately lighted by night, but some parts of the district were impossible for him for lack of illumination. This was the work he was unable to do. After his resignation he joined the panel, and by asking his patients whether, if he were unable to go to them, they would accept his partner this difficulty was overcome. Evidence that he was highly myopic was given, and the jury found that his incapacity was such as to render him unable to perform the duties of his office. Judgment was given that he was entitled to the superannuation allowance. It is no doubt irritating to any public body to see an official to whom they are paying a pension able to earn a living in another field, but the law rightly recognises that incapacity which applies to a previous office may not extend to other activities, and both medical men and guardians capable of taking an impartial view will congratulate Mr. J. D. Priest on his verdict.

### LORD WEMYSS ON HOMŒOPATHY.

As president of the London Homœopathic Hospital, Lord Wemyss, who is ninety-five years of age, sent a remarkable letter to excuse his absence at the annual meeting, in which he said: "I would gladly attend, but I am too crippled for such things. I, however, wish all success to homœopathy, to which I attribute my physical well-being in a great measure. When I was ninety I was asked to what I attributed my well-being at that late period of life, and my answer was, 'To parentage and moderation.' I should have added homœopathy, which I have practised since I was twenty." Lord Donoughmore, who presided, alluded to the new children's ward which Queen Alexandra has permitted to be named after her, and went on to say that while he was convinced that the claims of the hospital were fairly considered by King Edward's Hospital Fund—which has increased its grant from £400 to £600—he still adhered to the view that legacies should be put to the capital account, although the officials of the fund required them to be treated as income. It is interesting to note how certain peers of late have been identifying themselves prominently with various medical theories, and as far as homœopathy is concerned, at any rate, the hospitals professing it seem at no disadvantage in the public regard.

### DETERRENTS FROM SANATORIUM TREATMENT.

Speaking on the subject of the tuberculosis campaign, Dr. Penn Milton, medical superintendent of Devonshire Sanatorium, said that the important thing was to get hold of the predisposed children suffering from malnutrition, or convalescing from measles, whooping-cough, scarlet fever, and other childish ailments. Acting on this text, the Samuel Lewis Convalescent Home at Walton-on-the-Naze has decided to extend its work by placing in a house under its supervision a number of predisposed children from London. They stay there, we are informed, for a year, or even more, attend the local school, and are thus supervised perhaps at a turning-point in their physical development. In this connection a sanatorium correspondent writes: "There is a growing need for the education of the laity on the subject, and more especially of the employers in large workshops. There is at present a very real objection on their part to take back any employee who has been to a sanatorium, and this acts as a strong deterrent on the employee's side to seek sanatorium treatment at all. He is faced with the fear of losing his work on his return. This has, of course, been said before, but it is coming to be very fully realised that if an after-care committee is a vital part of the sanatorium benefit, the education of the employer will have to be a part of that branch's work." The sanatorium has probably been the most misrepresented of any class of institution from the days when everything was claimed for it to those in which it sometimes excites as much local opposition as isolation hospitals used to do. The after-care committees will, therefore, be doing useful work if they can educate the employer in the manner suggested by our correspondent. We should be glad to have the views of members of such committees on the subject.

### SYPHILIS DEPARTMENTS AT GENERAL HOSPITALS.

In the evidence given by Mr. D'Arcy Power, surgeon to St. Bartholomew's Hospital, at the twenty-second meeting of the Royal Commission on Venereal Diseases, last week, he laid stress on two points when discussing proposals for the best steps to be taken in combating and controlling the disease. In the first place, he said that he did not think it desirable that any organised attempt should be made to educate the public, but he put forward the proposal that every general hospital should establish a special department. There are signs that this last suggestion will be carried out, and the fact that Colonial institutions are now being followed by the London Hospital in establishing special wards indicates the probability of the wider adoption of this step. We cannot help feeling that the most hopeful general scheme yet publicly discussed was that described in The Hospital of September 27 last (page 749), when the Copenhagen system of consultation centres and impersonal notification was elaborated in detail. Of course, such a system must be supplemented by adequate and accessible means of treatment, sadly lacking in this country, and thus Mr. D'Arcy Power's suggestion is welcome as emphasising that feature of the desired programme which shows signs of being the first to be generally adopted.

### CAMBRIDGE MEDICAL SCHOOL AND STATE AID.

It seems doubtful if the heart-searchings caused by the proposed Government grant to the Medical School at Cambridge will be at an end now that the Grace in favour of an application for a grant was adopted by a majority of 33 last Saturday. State aid in the form of grants in aid are a recognised feature in almost every University; the Department of Astrophysics and the School of Agriculture at Cambridge itself are largely dependent upon them. But the constant scare of a Royal Commission, analogous in the world of the University to the invasion scare in the country as a whole, has whipped up all those forces which regard commerce between the University and the State as disastrous in principle and pregnant with State control. In the present case, moreover, the interval between the discussion of the proposal and the voting has been unusually short, so that there is, unfortunately, still room for difference of opinion to remain acute. The question to the world at large may seem of academic interest merely, but the Cambridge Medical School is of such repute as to make any prospective change in its financial or administrative arrangements worthy of note to institutional and medical men.

### TRADE TRIBUTE TO THE MIDDLESEX HOSPITAL.

Prince Alexander of Teck, chairman of the Middlesex Hospital, has received from Mr. Gordon Selfridge a cheque for £105, and also a letter testifying to the "effective aid which our own employees have repeatedly derived" from the institution. In the course of his letter the writer states that the cheque is a slight acknowledgment, on the completion of the fifth year of his business connection in London, of the hospital's services, and continues: "The great and populous trading community of Central London, the members of which, employers and employees alike, are bound together by a consciousness of the dignity of their work, lie under special and peculiar obligations to your hospital; and I may, perhaps, be permitted to say that it affords me, as a business man living amongst business men, the keenest pleasure to be of the least use in helping the Middlesex Hospital, which daily does so much to help us." The relations of hospitals with large firms, whether of the wholesale manufacturer or the retail salesman variety, have long been friendly, for men of business have early recognised that a subscription to a hospital is a very inexpensive form of insurance, being at the same time a graceful tribute to hospital work.

### A COURSE IN TROPICAL HYGIENE.

A course in tropical sanitation and hygiene will be a welcome addition to those conducted by the London School of Tropical Medicine. The first of the two courses annually which it is proposed now to hold will start on May 1, and the second on October 1, and each course will extend to eight weeks. The subject of "Tropical Sanitation and Hygiene" will be interpreted to include such important topics as the care of the sick on board ship in the tropics, port hygiene, surveying and sanitary engineering, medical entomology, bacteriology, and so on, and a great feature is to be made of practical demonstrations, so that the course may not be too theoretical a one. It is largely thanks to the success of the Chamberlain fund that means exist to start the new course, while, of course, the subjects of study have practically been the result of the work of Manson, Ross, and Bruce, to whom, it may be said, the Panama Canal is owing. The healthiness of the tropics for the white man is the magnificent goal of this branch of medicine, and it is only too probable that the importance of these regions to European nations is at present unrealisable, though pressure of population and the need for augmenting the food supplies of the Great Powers may bring it home to the man in the street by the end of the twentieth century. With tropical sanitation and hygiene taking academic rank among the medical courses a great step has been achieved.

### THE INSURANCE ACT AND "EXTRAVAGANT PRESCRIBING."

The deficiency in the drug funds in certain Insurance districts, which has been mentioned in the House of Commons, is a matter of some seriousness for the chemists in the affected areas. The causes of the deficiency have been considered at a conference of Lancashire Insurance Committees; but it is very doubtful whether the remedy suggested by the conference will have the desired effect. Briefly, it is proposed to recommend Insurance Committees to put in force the clause of the Medical Benefit Regulations which provides that deductions may be made from the fees due to panel doctors who, after an inquiry, are deemed to have prescribed extravagantly. It will surely be a very difficult task to decide what is extravagant prescribing. The district in which the largest deficiency seems to exist is Manchester, and in this case it would seem that the excessive call on the drug fund is due not to any extravagance in the nature of the drugs ordered, but rather to the large quantity of medicines consumed. In Manchester there are 262,700 insured persons, and the cost of drugs is something like £42,250; while in Liverpool the insured population numbers 247,120, and the drug bill is £22,000. While the number of prescriptions in Manchester is 1,281,771, the number in Liverpool is 634,000. That is to say, there has been double the amount of prescribing in Manchester, although there is not a vast difference between the insured population in the two cities. It is obvious that there is need for investigation, and everything points to the probability that the Manchester system of paying medical practitioners according to the number of attendances leads to more frequent prescribing.

### PROPOSED NEW HOSPITAL FOR DUNDEE.

The question whether or not Dundee should be provided with a children's hospital is continuing to excite much local interest. The high infantile mortality is advanced on the one hand, and the indebtedness of the existing Royal Infirmary on the other. The question is further complicated by two facts of great importance. Not only does the Royal Infirmary find considerable difficulty in raising the income which it needs, but it is unable at present to utilise all its beds; should, therefore, these be employed, in the event of better provision for children being made, or should any money raised be used to build a special children's hospital? That is the first practical difficulty once the question of providing institutional accommodation for the treatment of children is decided. The second is that the largest proportion of the children is said to be suffering from tuberculosis, and a feeling prevails that through the Local Government Board tubercular children may be dealt with and that the Government's attempts to treat tuberculosis in adults will not stop there. That being the state of public opinion in Dundee the practical course would seem to be to use the existing wish to provide better accommodation for children as a means of raising sufficient money to employ all the available accommodation at the Royal Infirmary, with the proviso that a certain specified number of beds should be set apart, structurally as well on paper, for children. In this way the existing movement would produce some, at least, of the results aimed at with promptitude and at little cost. The arrangement therein could await the double test of the practicability of including this work in that of the Royal Infirmary, and of the Government scheme above foreshadowed coming into being. In the meantime as the success of one side of hospital work never, experience teaches, otherwise than profitably affects other efforts, interest would be focussed on the Royal Infirmary to the advantage of that institution and of any children whom it was able to treat. How would such a proposal commend itself to the medical superintendent who may be expected to take an impartial view of what is practicable and desirable in this matter?

### MENTAL DEFECTIVES IN VICTORIA.

About two years ago a statistical inquiry was undertaken in Victoria with the object of finding the total number of mentally defective children in this State. Inquiries were made from State and private schools and from the medical profession, with the result that 2,500 were classed as "mentally dull," 860 as "feeble-minded," and 465 as idiots and imbeciles. The committee of inquiry has represented to the Victorian State Cabinet that the duty of educating and segregating this large body of nearly 3,000 persons is imperative. The suggestions embrace special schools for the mentally backward and defective; residential schools for those needing special care and for those from rural districts; the segregation of adults in special colonies, and special legislation dealing with segregation. Dr. Ernest Jones, Inspector-General of the Insane, is preparing a Bill on the subject for the State Cabinet.

### THE SUB-DIVISION OF HOSPITAL WORK.

When we turn to an examination of the economical and administrative departments of a great hospital a revolution in the old methods of management and organisation is seen to have taken place. To-day every reputable hospital which is properly administered keeps its books upon the Uniform System. The economic control is extended to the minutest detail as it may affect each and every department throughout the whole establishment. The secretary's and superintendent's section, in the best of hospitals, is an elaborately organised and attractively developed business, often staffed by men of the highest intelligence and the most careful technical training. Inquirers who have a right to ask questions are welcome, and their satisfaction at the results of any tests they may apply to the business of a particular hospital through the secretary is the measure of the inquirer's competence to form a sound judgment out of great practical knowledge. Take the matron's department, and write down the names of the various sections into which this branch of the work is now divided up. Spend half a day in going through these departments, in seeing the workers at work, and in getting to know exactly what is being attempted, and what are the fruits which women now produce in a great hospital. We venture to say that in the nursing, the wards, the preliminary and continuous training of the probationers, the economy, management and output of the laundry, the organisation of the matron's office, the practical aid rendered by sisters in the out-patient, massage, light, casualty, and many other portions of the establishment the results are so good as to make one thank God and take courage.

### OUR PRIZE SCHEME FOR EQUIPMENT "TIPS."

While the voluntary system and the associations of hospital officers have produced a ready means of interchanging views and important experience among the different institutions, what may be called the smaller "tips" of institutional equipment and economy are still too much the secret of isolated departments, which rarely permeate from one hospital to another. Yet the need for such "tips" is great, as is shown by the numerous inquiries which we have received concerning the description of the Cromer ward-locker, which appeared in the Institutional Worker section of The Hospital in November last. We have therefore decided to offer two prizes, one of five guineas and the other of two guineas, for the description of the best appliance in actual use, or of a suggested improvement, which shall be sent to us on or before April 18. Every class of institutional appliance and requisite will form the subject of a prize competition in due course, so that there may be an inducement for institutions possessing some contrivance of which they are especially proud (and the smallest cottage hospital has one!) to share their advantage with others through the medium which the pages of The Hospital afford. Full particulars of the prize scheme, which contains some novel features, will be found on page xli of our advertisement columns. The first article chosen for description is the "ward-locker," since the demand for a first-class design is known to us to be large, in spite of the many patterns on, or, as in some of the best hand-made devices, not on the market. In this way all the best types of furniture, appliance, and contrivances for securing economy should be rescued from oblivion and the smaller points of practical efficiency, equally with the larger, be made common to the whole institutional world. To secure complete success it remains for those institutions *in need* of some requirement also to let us know, so that every article for which there is a demand, or in which an improvement is sought, may be promptly offered for competitive description.

### THE SUPPLY OF COD-LIVER OIL.

Those who have read the weekly reports on the Drug Market which appear in these columns will have observed that during the past few weeks the value of cod-liver oil has been steadily declining. Quotations have now reached a figure which might appear tempting to those who buy cod-liver oil in large quantities, and as the drug is an important one, and the present state of the market of exceptional interest, the position is worthy of discussing in a separate note. When the market for the new season's oil opened about the end of January, the price asked was a few shillings above that at which last season's oil had been slowly selling. As the fishing proceeded, however, and the weather improved, it became obvious that the catch was likely to be a good one and the yield of oil plentiful. Compared with an even date last season the yield of cod-liver oil up to the present this season has been very much larger, and if good weather continues to prevail a fairly abundant supply of cod-liver oil will be available. Present quotations are something like 30 per cent. lower than those quoted at the opening of the fishing season, and although prices have been lower they have frequently been very much higher. It is not easy to decide what is the best course for buyers to take, but there appears to be no reason to go on to the market immediately. The trend of the market, however, should be watched very carefully, and it may seem advisable to make purchases quite soon. Incidentally it may be added that the consumption of cod-liver oil in this country has been considerably increased by the operation of the Insurance Act; but, while this increase might be an important factor in a season of small supplies, its effect will perhaps not be considerable in a plentiful market.

### THIS WEEK'S DRUG MARKET.

The volume of business has not been large during the week, but there has been a fair demand for drugs which are in common request at this season of the year. Ipecacuanha has been sold at higher rates, the larger demand which has of late been evinced being due to the increasing use of emetine, the chief alkaloid of ipecacuanha, in the treatment of dysentery and other tropical diseases. Opium is quiet, but its value is well maintained; morphine continues to be in fair request at steady rates. American oil of peppermint is again rather dearer, but Japanese dementholised oil is dull. Menthol is barely steady. Citric acid is dearer in consequence of shortage of supplies, and makers of citrates have advanced their prices accordingly. The demand for cocaine continues very quiet, notwithstanding the low prices at which it is obtainable. Ergot of rye has an upward tendency in price. The value of essence of lemon is declining. The value of belladonna is well maintained. There is a fair demand for camphor at steady rates. Cod-liver oil has continued to decline on reports of successful fishing operations in Norway; business has not been on a large scale, the general impression being that prices have not reached the lowest.

### TO OUR READERS.

Contributions are specially invited from any of our readers to these columns. They should deal with topical subjects and news. They must be authenticated for the information of the Editor only. The minimum payment if published is 5s. There is no hard-and-fast rule as to space, but notes of about twenty lines in length are preferred.

